# Incorporating informatively collected laboratory data from EHR in clinical prediction models

**DOI:** 10.1186/s12911-024-02612-1

**Published:** 2024-07-24

**Authors:** Minghui Sun, Matthew M. Engelhard, Armando D. Bedoya, Benjamin A. Goldstein

**Affiliations:** 1 Department of Biostatistics and Bioinformatics, Duke University, Durham, NC USA; 2 Department of Medicine, Duke University, Durham, NC USA

**Keywords:** Electronic Health Records, Missing Data, Embedding, Machine Learning

## Abstract

**Background:**

Electronic Health Records (EHR) are widely used to develop clinical prediction models (CPMs). However, one of the challenges is that there is often a degree of informative missing data. For example, laboratory measures are typically taken when a clinician is concerned that there is a need. When data are the so-called Not Missing at Random (NMAR), analytic strategies based on other missingness mechanisms are inappropriate. In this work, we seek to compare the impact of different strategies for handling missing data on CPMs performance.

**Methods:**

We considered a predictive model for rapid inpatient deterioration as an exemplar implementation. This model incorporated twelve laboratory measures with varying levels of missingness. Five labs had missingness rate levels around 50%, and the other seven had missingness levels around 90%. We included them based on the belief that their missingness status can be highly informational for the prediction. In our study, we explicitly compared the various missing data strategies: mean imputation, normal-value imputation, conditional imputation, categorical encoding, and missingness embeddings. Some of these were also combined with the last observation carried forward (LOCF). We implemented logistic LASSO regression, multilayer perceptron (MLP), and long short-term memory (LSTM) models as the downstream classifiers. We compared the AUROC of testing data and used bootstrapping to construct 95% confidence intervals.

**Results:**

We had 105,198 inpatient encounters, with 4.7% having experienced the deterioration outcome of interest. LSTM models generally outperformed other cross-sectional models, where embedding approaches and categorical encoding yielded the best results. For the cross-sectional models, normal-value imputation with LOCF generated the best results.

**Conclusion:**

Strategies that accounted for the possibility of NMAR missing data yielded better model performance than those did not. The embedding method had an advantage as it did not require prior clinical knowledge. Using LOCF could enhance the performance of cross-sectional models but have countereffects in LSTM models.

## Introduction

Electronic Health Record (EHR) data are widely used to develop clinical prediction models (CPMs). The greatest strength and weakness of using EHR data for developing such tools is that these data reflect the way information flow in a real-world environment. On the positive side, data used to create the models represent how patients interact with the healthcare systems and thus share the same characteristics as the data available later during model deployment [1–3]. On the downside, the way of collecting clinical information is not uniform across patients, providers, and clinical services. The inconsistency leads to varied rates of unobserved (or missing) clinical measures. More importantly, the missingness is typically highly informative with the generating mechanism not observable. Therefore, addressing missingness in EHR can be a challenging problem [1–5].

When developing and ultimately implementing CPMs, it is necessary to consider how best to handle missingness among the predictor variables. A variety of strategies have been suggested for addressing missing data issues in predictive modeling, ranging from complete-case analysis to machine learning methods and to various forms of imputation strategies [6–9]. However, when we choose a missing data procedure, it is crucial to consider and account for the underlying missingness mechanism. In inferential statistics, a lot of work has been conducted in characterizing the type of missingness. The most widely used framework is put forward by Little and Rubin [10]. In this framework, mechanisms dictating the missingness patterns are recognized as Missing Completely at Random (MCAR), Missing at Random (MAR), and Not Missing at Random (NMAR), described in more details in the next section. Of note, within this framework, different approaches are appropriate based on the underlying mechanism.

CPMs based on time-varying longitudinal data present some unique challenges and opportunities with regards to missing data. When risk assessments are made serially overtime, there are more opportunities for data not to be observed. However, one can also leverage the longitudinal nature of the data to impute in or model the missingness.

The goal of this paper is to explore the suitability of different approaches for handling missing data in the context of developing a longitudinal CPM when data are informatively missing. We take a case study in building a predictive model for inpatient deterioration, for which we have previously developed and implemented into our EHR system [11]. In particular, our CPM utilized laboratory tests that were only collected when a clinician determined they were of values to measure. We first give a formal presentation of missing data within the Little and Rubin [10] framework. We next discuss different strategies for handling missing data. Then, we describe the data in our case study along with our implemented analytic strategies, followed by the results of our empirical evaluation. We finally conclude by discussing the implications of our findings.

## Materials and methods

### Missingness mechanisms

We consider a scenario where the dataset of interest contains missing values. We use a matrix of Bernoulli random variables to represent the missingness. Each item in the matrix indicates the missingness state of the corresponding entry in the original data matrix. The matrix, dictated by a missingness mechanism, is a part of the data generating process. This mechanism plays a pivotal role in the study of missing data analysis [12]. It helps to characterize the relationship between the missingness matrix and the original data matrix. The mechanism applies to both outcome and input data in a supervised learning context. However, in this paper, we only consider scenarios where there is missingness amongst the predictor variables.

According to Little and Rubin [10], there exist three types of mechanisms: Missing Completely at Random (MCAR), Missing at Random (MAR), and Not Missing at Random (NMAR). Following their notation, we denote $\:\mathbf{X}$ as the input data matrix. For an arbitrary observation, the input data $\:\mathbf{x}$ consists of two parts: $\:{\mathbf{x}}_{\text{mis}}$, the missing variables, and $\:{\mathbf{x}}_{\text{obs}}$, the observed variables. We denote $\:\mathbf{M}$ as the missingness matrix, where

$$\:{M}_{ij}=\left\{\begin{array}{l}1,\hspace{0.25em}\hspace{0.25em}\hspace{0.25em}\text{if\:the\:valueis\:missing}\\\:0,\hspace{0.25em}\hspace{0.25em}\hspace{0.25em}\text{otherwise}\end{array}\right.$$

We also let $\:\mathbf{m}\in\:\{0,1{\}}^{p}$ indicate the observation-wise missingness state, where $\:p$ is the number of predictors in the input data. The mechanism is categorized by a conditional distribution, $\:f\left(\mathbf{m}|\mathbf{x},\varvec{\Theta\:}\right)$, where $\:\varvec{\Theta\:}$ is an unknown parameter vector that defines input variables. In the definitions below, we combined Little and Rubin’s probability density representation [10] and the explanation proposed by Sterne et al. [13].

#### Definition 2.1.1

In MCAR, the probability of being observed is independent of any variable. That is, $f(m|x,\Theta ) = f(m|\Theta )$. Equivalently, within each variable with missingness, there is no systematic difference between missing and observed values. As an illustration, this may occur if some lab measurements are unable to be collected due to a backlog in the lab.

#### Definition 2.1.2

In MAR, the likelihood of being observed can only depend on the observed variables. That is, $f(m|x,\Theta ) = f(m|{x_{obs}},\Theta )$. Equivalently, within each variable with missingness, the difference between missing and observed values can be explicitly explained by other observed variables. This may happen when sicker patients have more measurement, and the missingness is attributable to completely observed health status variables.

#### Definition 2.1.3

In NMAR, the likelihood of being observed can depend on both the observed and the missing variables. That is, $f(m|x,\Theta ) = f(m|{x_{obs}},{x_{mis}},\Theta )$. Equivalently, within each variable with missingness, the systematic difference still remains between missing and observed values even if we adjust for other observed variables. This may happen in the case where sicker patients have more measurement, but the health status indicators are not recorded.

NMAR is also known as informative missingness [14]. This commonly occurs with EHR data, where lab measurements are obtained from the patients only when doctors believe they are necessary. For example, as previously described, sicker patients tend to have more clinical measurements and, consequently, more data within the EHR than healthier patients [8]. This has also been referred as informed presence bias, the notion that what we observed from EHR data is inherently informative [1]. For instance, let us consider a scenario where we want to build a classification model for predicting cardiovascular disease among patients. Then, many healthy patients may have missing cholesterol data since doctors saw no need for the measurement [15]. Moreover, the informative missingness contributes to the outcome prediction [16].

Overall, missing data is a common challenge for developing CPMs but has not been well treated in many studies. A recent review of handling missing data for CPMs [6] found that only about 60% of searched studies reported strategies for addressing missing data, and complete-case analysis was the most frequently used approach among them. Other methods, such as multiple imputation (MI), k-nearest neighbor imputation, mean imputation, and indicator variable methods, were implemented in a limited number of cases. Another review of CPMs using EHR data, found 44% of studies failed to report any approach to handling missing data. Of those that did report an approach, imputation methods (single and multiple) were most commonly used [17].

### Current study

#### EHR data

For our study, we consider the problem of developing a predictive model for inpatient deterioration. We have previously reported on how we developed and implemented such an early warning score (EWS) at our institution [11]. The EWS uses real-time patient information on demographics, comorbidities, vital signs, and laboratory measurements. Of particular interest are the laboratory measurements, which are only measured when clinicians deem them necessary, so the missingness is likely informative. In the original model, we used a categorical imputation approach as described above. Here we further explore the performance of alternative strategies.

##### Data source

We use the original source data from when we developed the EWS. The data include all inpatient adult (age 18) admissions to a general medical or surgical unit at Duke University Hospital – a 957-bed, tertiary care, academic medical center – from 2014 to 2016. Data were extracted from EPIC based EHR system. The study was approved by Duke Health IRB at Duke University with the IRB number Pro00060340. Participant consent was waived for this retrospective analysis due to the minimal risk posed to study participants and the infeasibility of obtaining consent in a large retrospective cohort.

##### Data set-up

The operationalized score operates as a triage score. Nurse managers use the score at 8 am and 8 pm daily to identify patients at risk of deteriorating in the next 12 h. As such, we set our data up to reflect this use case. Specifically, we organized patient data into 12-hour blocks. As in our original model development, patient time was censored after 7-days since events are relatively rare after this point. As such, each patient had at most fourteen time-blocks. The data for each time block are based on what was measured in the previous 12 h.

##### Outcome of interest

The outcome of interest is rapid deterioration. This is defined as a transfer from a general medical or surgical floor to an intensive care unit (ICU) or mortality while on a general medical or surgical floor within the first week since admission. Heuristically, this outcome can be thought of as someone who is in a relatively stable condition and then has an adverse outcome. To define the outcome, we extracted time-stamped unit information to identify when patients were on a unit of interest and had the event of interest. The outcome was a modeled as a binary outcome of deterioration within the following 12-hour blocks.

##### Predictor variables

**Fig. 1:**
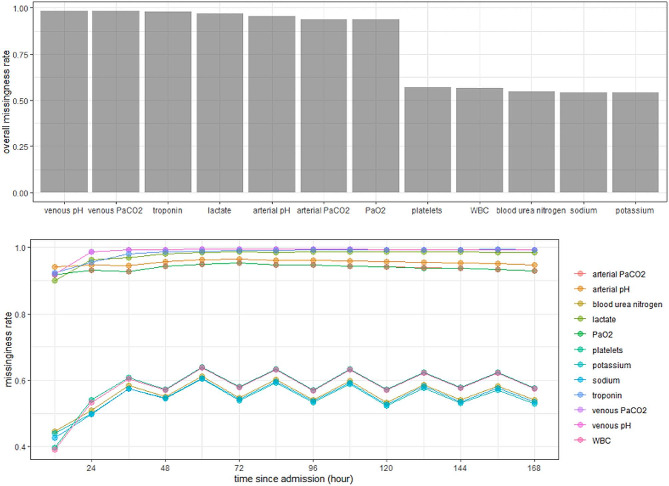
Missingness rate of the laboratory measurements

For this study, we focused on predictor variables from the original CPM, which had potential informative missingness, such as the laboratory measures. In consultation with our clinical collaborator (ADB), we identified five laboratory tests that should be regularly collected from all patients (white blood cell count, platelet, sodium, potassium, blood urea nitrogen) and seven laboratory tests that would be collected more informatively (arterial and venous pH, PaO2, arterial and venous PaCo2, lactate and troponin). Figure 1 shows the missingness rate for all of the laboratory measures. Additionally, for modeling purposes we incorporated demographic factors of age, sex, and race.

#### Missing data strategies

In this section, we discuss the different missing data strategies we considered. Each method relies on different assumptions regarding the missingness mechanism.

##### Mean imputation

In mean imputation, all missing attributes are estimated by the average of the corresponding ones from the observed subjects. Mathematically, the process can be expressed as follows.

$$\left\{\begin{array}{l}\forall \: j \in \{ 1, \ldots ,p\} \\N_{\text{obs}_j} = \sum\limits_{i = 1}^N \left( 1 - M_{ij} \right) \\\hat{x}_j = \frac{1}{N_{\text{obs}_j}} \sum\limits_{i = 1}^N \left( 1 - M_{ij} \right) X_{ij}\end{array}\right.$$

where $\:{\widehat{x}}_{j}$ is the imputed value for the $\:j$-th predictor. When the missingness mechanism is MCAR, this approach preserves the variable means but can distort the correlation structure among predictors [12].

##### Conditional imputation

Conditional imputation fills in the missing data with random draws from predictive distributions that depend on the observed values from other predictor variables. We use predictive mean matching as the conditional model to estimate the missing values. All the predictors are included in the conditional model. Predictive mean matching is less vulnerable to model misspecification than linear regression and thus has high robustness [18]. We perform the chain equation algorithm to achieve convergence in the estimated values and set the number of iterations as 10 based on the rule of thumb [19]. This approach assumes MAR, which may introduce bias to the data when the missingness is informative [19].

##### Normal-value imputation

In the normal-value imputation method, missing components are replaced with the expected normal values, which are calculated as the average of the lower and upper bounds of the normal range provided in Table 1. These range values are determined by medical professionals and healthcare practitioners. This imputation approach assumes that a laboratory measurement is not taken because it is presumed to fall within the normal range. Consequently, this method operates properly only when the assumption holds true, which is a special NMAR mechanism.

**Table 1:** Range and normal range of each laboratory measurement

Lab	Lower limit	Lower limit of normal range	Upper limit of normal range	Upper limit
BUN	0.00	7.00	20.00	150.00
Potassium	0.00	3.50	5.00	14.00
Platelets	0.00	150.00	450.00	3000.00
Sodium	85.00	135.00	145.00	200.00
WBC	0.00	3.20	9.80	500.00
pH (aterial)	6.75	7.35	7.45	7.65
pH (venous)	6.75	7.32	7.42	7.65
PaO2	20.00	75.00	100.00	670.00
PaCO2 (aterial)	5.00	35.00	45.00	200.00
PaCO2 (venous)	5.00	39.00	55.00	200.00
Lactate	0.00	0.50	2.20	20.00
Troponin	0.00	0.00	0.10	40.00

##### Categorical encoding imputation

Based on the prior knowledge about the lab predictor variables, we categorize each of them into four levels, including “unknown,” “normal,” “high,” and “low” [11]. Table 1 provides all the cut points for the categorization of each lab measurement. The categorization completes the original data. It allows us to fit a classification model to informative missingness. Moreover, it can capture the potential nonlinear relationships (e.g., U-like relationships) between the predictors and the target. This method does not make any assumption about the missingness mechanism. However, it requires substantial background knowledge about data. Furthermore, categorizing continuous data without scientific reason may worsen classification models’ performance and efficiency [20].

##### Last observation carried forward (LOCF)

For each of the above methods, we also consider the use of LOCF (also known as forward-filling or forward imputation). LOCF is a common method applied to longitudinal data [21]. All missing values are filled by the last observed values of the corresponding variables. Since it places a strict time-dependent structure on the data, it has theoretical soundness under limited scenarios of MAR [22].

##### Missingness embedding

All of the methods described above fill in the blanks of the input data matrix. They are all feasible for any kinds of predictive modelling techniques. However, in the context of deep learning, we can directly incorporate the missingness into the modelling approaches instead of separately completing the input data.

Consider a partially observed variable, $\:{x}_{j}$, from $\:\mathbf{x}$ with missingness status $\:{m}_{j}$ from $\:\mathbf{m}$. Let’s assume a generalizable $\:k$ represents the number of categories for missingness status. Next, let $\:{\mathbf{m}}_{j}\in\:\{0,1{\}}^{k}$ be the one-hot representation, also known as a standard basis vector, where it has 1 at position $\:k$ and 0 at all other entries. Additionally, let $\:{W}_{j}\in\:{\mathbb{R}}^{k\times\:d}$ be the embedding matrix, where $\:d$ is the dimension of the resultant embedding vector. The embedding representation, $\:{\mathbf{e}}_{j}\in\:{\mathbb{R}}^{d}$, is obtained by $\:{\mathbf{e}}_{j}={W}_{j}{\mathbf{m}}_{j}$ [23, 24]. We also use a conditional scaling technique to incorporate all the observed values. If $\:{m}_{j}=1$, $\:{\mathbf{e}}_{j}$ will remain unchanged; otherwise, $\:{m}_{j}=0$, the resultant embedding will be $\:{x}_{j}{\mathbf{e}}_{j}$ [24]. In this approach, the transformation only happens to variables with missingness, while fully observed variables is unaffected. By mapping the binary indicator variable to a higher-dimensional embedding space, this method can uncover intrinsic properties related to the missingness status of the variable [23]. The dimension of the embedding, $\:d$, is a hyperparameter that may vary across different variables. According to the rule of thumb, its size can be $\:\sqrt[4]{k}$ [25]. For example, in our case, as we had two missingness states (where $\:k=2$), we selected the dimension $\:d$ as 2 by using the formula and rounding up the result to the next whole number.

**Fig. 2:**
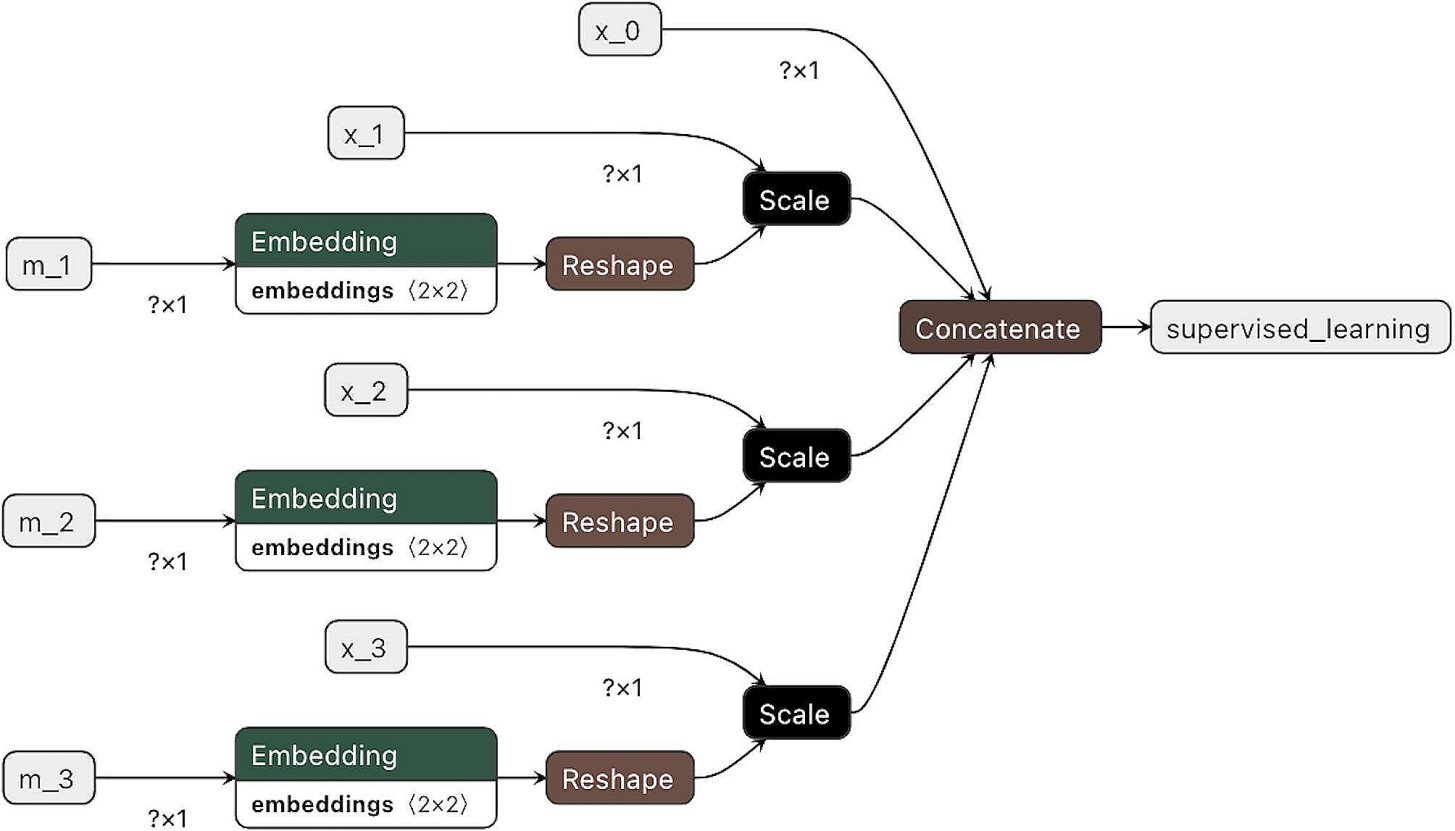
An example of missing embedding

Embedding weight matrices $\:{W}_{j}$’s are tuned along with the training of a downstream deep learning model. The weight matrices transform the original incomplete dataset into a complete concatenation of embeddings. The transformed dataset carries all the information about the observed raw values and informative missingness. Figure 2 visualizes and exemplifies how this approach is implemented when there are three predictors with missingness and one fully observed predictor.

Built upon different missingness mechanisms, missing data strategies are case-specific. Table 2 summarizes the various methods and the missingness mechanism types they can rely on.

**Table 2:** Missing data strategies and the mechanisms in which they are valid to use

method	mechanism
Mean imputation	MCAR
Conditional imputation	MCAR/MAR
Normal-value imputation	NMAR^a^
Categorical encoding	MCAR/MAR/NMAR
LOCF	MCAR/MAR^b^
Missingness embedding	MCAR/MAR/NMAR

^a^Normal-value is valid only under our assumption on the informative missingness and not generalizable to all NMAR settings

^b^LOCF works the best when the basis value and forward-imputed value share the same distribution

#### Classification models

We implemented three different types of classifiers through which to compare the missing data methods. We used logistic LASSO regression, multilayer perceptron (MLP) and recurrent neural network using long short-term memory (LSTM). For the logistic LASSO regression and MLP, we modeled the data cross-sectionally, ignoring the repeated measurement nature of the data. For the LSTM, we explicitly accounted for the longitudinal data structure.

#### Implementation details

We divided our data into training (80%) and testing (20%) data, splitting at the patient level. For the data-dependent strategies (e.g., mean imputation, conditional imputation and embedding), we first learned the imputation strategies and predictive models from the training data. Subsequently, we applied both the strategies and the models to the testing data.

We applied each of the four general imputation methods (excepting the embedding approach) both with and without LOCF and fitted each of the three models. Additionally, we applied the embedding approach only under the MLP and LSTM models. Moreover, given that the embedding framework can be applied to any categorical data (e.g., sex, race, etc.), we investigated if embedding encoding the categorical variables could perform differently from one-hot encoding them. Thus, we implemented the missingness embedding methods for both scenarios to make a comparison. Despite the categorical predictors’ varied levels, we made all embedding dimensions the same for simplicity.

For the logistic LASSO regression models, we used 10-fold cross-validation with grid search to optimize the penalizing coefficient $\:\lambda\:$. For the neural network models, we used Hyperband [26], a reinforcement-learning-based random search algorithm, to navigate towards the optimal neural architecture. This framework allows the models to automatically learn hyperparameters, but there is a trade-off between computing efficiency and optimality [26]. Two additional parameters were introduced to manage the trade-off: the maximum number of epochs a trial can run and the trial eliminating factor. Li et al. [26] provided comprehensive guide for setting their values. Neural network models’ hyperparameters, including model number of hidden layers, number of neurons per layer, dropout rate, and learning rate, were all optimized by this framework.

#### Data and model evaluation

We described the patient cohort based on those who experienced rapid deterioration (the case group) versus those who did not (the control group). To assess the potential for informative missingness we investigate whether the case group had more measurements of each lab compared to the control group. We used different methods to compare cases and controls for the two distinct categories of laboratory measurements. For labs that were regularly measured and more frequently observed (e.g., white blood cell count), we used a count-based quasi-Poisson regression with the length of stay as the offset. For the infrequently but informatively measured ones (e.g., lactate), we used an ever/never relative rate regression via a Poisson regression with a sandwich estimator, while also controlling for the length of stay [27]. We calculated relative risks (RR) and the corresponding 95% confidence interval (CI) as the metrics for comparison.

We evaluated the fitted models on the test data based on area under the receiver operator characteristic (AUROC). To account for the longitudinal data’s intra-subject correlation within model evaluation, we calculated a weighted average of AUROC stratified by the length of stay, where observations were independent within each stratum. The weights were the observation-level proportions of length of stay values. Additionally, we used a basic bootstrapping algorithm to calculate the 95% CIs, where observations were resampled at the patient level.

The logistic LASSO regression models were fitted in R 4.1.2 [28] using glmnet [29]. The MLP and LSTM models were fitted in Python 3.10.4 [30] using TensorFlow 2.13.0 [31].

## Results

### Data summary and informative missingness

There were 105,198 patients. After bucketing the data into 12-hour time blocks, we obtained 870,869 observation units for this study. Within the studied time window, patients had on median 4 hospital days (IQR: 2 to 7) corresponding to about 8 observation points per person. The overall patient-level event rate was 4.7%, with an observation-level event rate of 0.6%. Table 3 reports demographic characteristics of the studied patients.

**Table 3:** Comparison of baseline demographic information between case and control groups

	Control (*N* = 100,187)	Case (*N* = 5011)	SMD^a^
**Length of Stay (hour)**			0.0502
Median [Q1, Q3]	96.0 [48.0, 168]	96.0 [54.0, 144]	
**Admission type**			0.504
Elective	29,015 (29.0%)	2567 (51.2%)	
Emergency	44,193 (44.1%)	1795 (35.8%)	
Trauma Center	14 (0.0%)	1 (0.0%)	
Urgent	26,965 (26.9%)	648 (12.9%)	
**Sex**			0.184
Female	53,777 (53.7%)	2230 (44.5%)	
Male	46,410 (46.3%)	2781 (55.5%)	
**Age (year)**			0.366
Median [Q1, Q3]	58.0 [40.0,69.0]	64.0 [52.0, 73.0]	
**Race**			0.307
American Indian or Alaskan Native	649 (0.6%)	40 (0.8%)	
Asian	1729 (1.7%)	58 (1.2%)	
Black or African American	30,248 (30.2%)	967 (19.3%)	
Caucasian/White	61,591 (61.5%)	3752 (74.9%)	
Missing	1594 (1.6%)	86 (1.7%)	
Other	4376 (4.4%)	108 (2.2%)	

^a^The standardized mean difference (SMD) is utilized as the metric for comparing the groups

Table 4 presents and compares the rates of observing each laboratory measurement. Notably, the missingness rates exhibit significant differences between controls and cases across all labs, suggesting the presence of potentially informative missingness.

**Table 4:** Comparison of the degree of missingness in laboratory measurements between case and control groups

		Control (*N* = 100,187)	Case (*N* = 5011)	RR (95%CI)
**Regular**:				
**Median [Q1**, **Q3]**^**a**^				
	BUN	3.00 [1.00,6.00]	4.00 [2.00,7.00]	1.25 (1.24,1.27)
	Potassium	3.00 [1.00,6.00]	5.00 [2.00,7.00]	1.27 (1.26,1.29)
	Platelets	3.00 [1.00,5.00]	4.00 [2.00,6.00]	1.25 (1.23,1.27)
	Sodium	3.00 [1.00,6.00]	5.00 [2.00,7.00]	1.27 (1.26,1.29)
	WBC	3.00 [1.00,5.00]	4.00 [2.00,6.00]	1.25 (1.23,1.26)
**Informative**:				
**Observing rate** ^**b**^				
	pH (arterial)	11.75%	52.88%	5.00 (4.84,5.16)
	pH (venous)	9.74%	16.86%	1.80 (1.69,1.93)
	PaO2	15.83%	70.78%	4.97 (4.84,5.10)
	PaCO2 (aterial )	15.82%	70.74%	4.97 (4.84,5.10)
	PaCO2 (venous)	9.76%	17.06%	1.82 (1.71,1.94)
	Lactate	14.47%	39.99%	2.96 (2.85,3.08)
	Troponin	10.41%	19.06%	1.90 (1.79,2.02)

^a^The median count, with 1st and 3rd quartiles, of observed values for a lab among all patients

^b^The probability of the lab ever being observed from a patient

### Evaluation of method performance

The baseline models which did not include any laboratory variables achieved the following aggregated AUROC scores with the corresponding 95% confidence intervals: logistic LASSO regression 0.64 (0.623, 0.658), MLP had 0.715 (0.696, 0.733), and LSTM had 0.732 (0.713, 0.751). We then used them as a reference and examined how the incomplete laboratory measures could improve the model performance and how different missing data strategies could affect model performance.

**Fig. 3:**
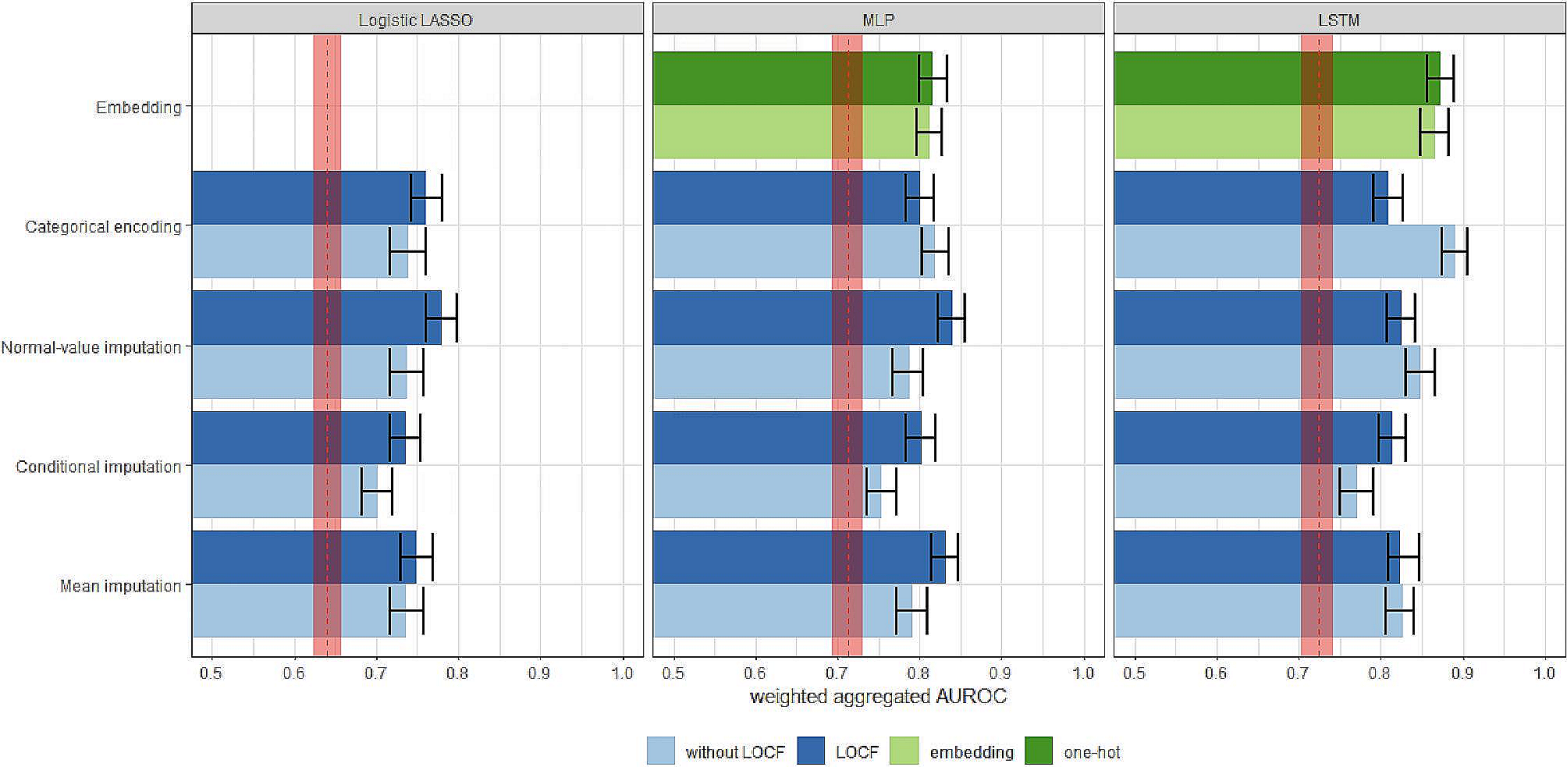
Discriminative capability of the full model using various missing data strategies. Red dashed lines and shaded areas represent benchmark scores obtained in the absence of lab measures and their corresponding 95% CIs

Figure 3 summarizes the performance of each classifier using each missing data approaches on the test data. The error bars on the plot represent the 95% CIs for the corresponding metric. The red dashed lines and shadows are the scores and 95% CIs of the baseline models. We observed that all three classifiers generally performed better after incorporating the twelve laboratory measures and addressing the missingness. Additionally, the LSTM model, which accounts for the longitudinal structure of the predictors, had the best overall aggregated AUROC, followed by the MLP and logistic LASSO regression.

For cross-sectional models using logistic regression or MLP, LOCF generally improved the imputation methods, with some improvements statistically significant. The conditional imputation had the worst performance across all classifiers. Normal-value imputation with LOCF was the best strategy for both cross-sectional models.

Results were different for the LSTM, which changed the missing data strategies’ performance pattern. Missingness embedding and categorical encoding both performed the best. Furthermore, LOCF led to significantly reduced performance for categorical encoding and a slight but insignificant drop in normal-value and mean imputations.

**Fig. 4:**
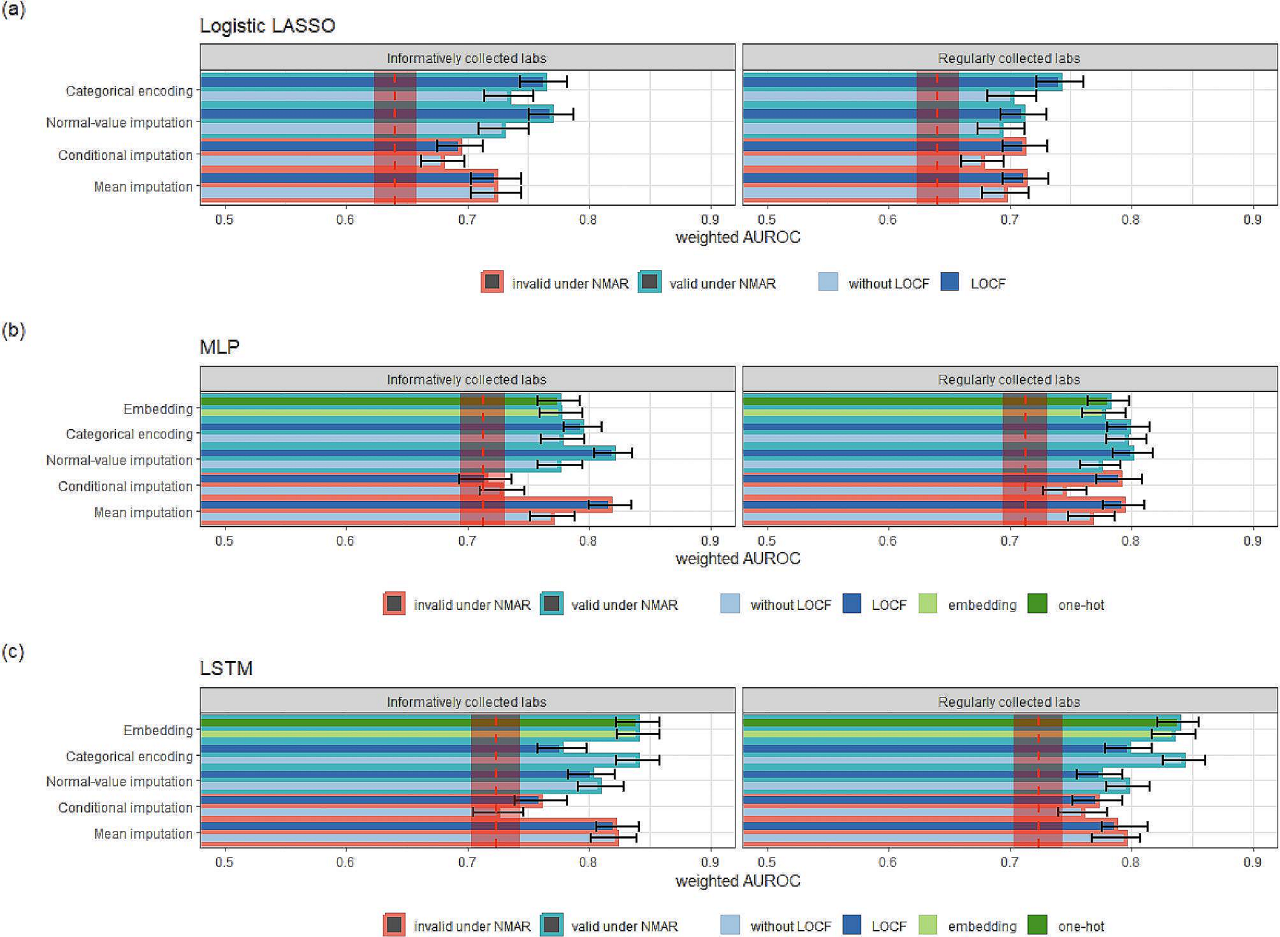
Discriminative capability of the reduced model using various missing data strategies. Red dashed lines and shaded areas represent benchmark scores obtained in the absence of lab measures and their corresponding 95% CIs

Figure 4 summarized how different methods performed when only considering two subsets of the lab variables: one for the regularly observed variables and the other for the informatively collected variables. The reduced models retained some observations from the full model, and we found that LSTM models generally outperformed cross-sectional models. LOCF was generally beneficial for imputation strategies in cross-sectional models, but it tended to reduce performance in LSTM models. However, there were some additional insights to be gained.

Parts (a) and (b) showed that for cross-sectional models, categorical encoding with LOCF tended to perform best for regularly collected labs, while normal-value imputation with LOCF tended to be the best for informatively collected labs. Part (c) demonstrated that missingness embedding and categorical encoding were more effective than other strategies when applied to regularly collected labs.

## Discussion

### Insights from the results

Our study explored five methods for addressing informative missing when predicting inpatient deterioration using EHR data. We compared their performance using three classification models: logistic LASSO regression, MLP, and LSTM. One advantageous aspect of predictive modeling is that, when handled appropriately, missing data strategies can be treated as tunable parameters that can be learned during the model development process.

The learning task was based on EHR data with informative missingness. Applying mean imputation and conditional imputation, as well as their LOCF augmented versions, was theoretically unsuitable. We compared them to methods that followed the informative missingness assumption. Overall results showed it was critical to employ a valid missing data strategy with a longitudinal downstream model to predict inpatient deterioration.

Missingness embedding is an advanced indicator variable method that uses scaled embedding vectors from $\:{\mathbb{R}}^{d}$ to express observed values and missingness, rather than binary values [24]. During the training process, embedding vectors are continuously learned, tuned, and optimized by the data. Unlike an ordinary indicator variable method that still would require an additional imputation method to complete the dataset, missingness embedding was self-sufficient. This means that models could be directly fitted using the available data without requiring imputation. Moreover, missingness embedding is integrated to prediction models by its design, which makes the model ready for immediate deployment. In contrast, models trained using data processed by most imputation methods typically required complete data during deployment, as the imputation models are usually not integrated with the prediction models [3].

Compared to one-hot encoding, using embeddings to encode categorical demographic variables did not improve the performance in our study. Presumably, the embeddings would reveal the categorical variables’ intrinsic properties and thus help the classifier solve the prediction task [23]. It has been verified in various cases where the embedding representation of categorical variables with hundreds or even higher numbers of levels was powerful and advantageous [32–34]. However, in our specific case, we did not observe any improvement using embedding representations. This can be attributed to the fact that we had an enormous number of observations where all the categorical variables only have a few types. One-hot encoding them would not introduce any sparsity.

As expected, predictive performance improved with increasing algorithm complexity. Laboratory values typically have complex, non-linear effects, for which a standard LASSO regression cannot adequately model. Moreover, an LSTM can capture more longitudinal patterns. Therefore, it is not surprising that the optimal missing data strategy varied based on model type. The categorical imputation had the greatest impact when using the LASSO, likely because it allows for a non-linear representation of the data. That effect is less pronounced when using the deep learning methods.

When using a cross-sectional model (i.e., LASSO or MLP), LOCF was typically optimal, particularly when combined with normal-value imputation. Conversely, with the longitudinal model, (i.e., LSTM), LOCF was not preferred. A reasonable rationale could be LOCF set the data into a temporal structure. After combining with the normal-value imputation, it generated a complete dataset that followed the assumption of informative missingness that values were taken only when needed. Moreover, in Fig. 4 (b), we saw it significantly boost the invalid strategies when the model only incorporated the regularly collected labs. However, LOCF has been critiqued for casting a strict ideal structure on data [22]. This could explain why LOCF generally reduced the performance in LSTM models, which were designed to automatically learn the time correlation in the data. Putting restricted but incorrect format on the time-varying information would therefore harm the classifiers’ discriminative capability. Especially, when LOCF was combined with categorical encoding, the performance was significantly reduced.

If we only incorporated a subset of all the laboratory variables, the model performance decreased throughout all missing data methods and classification models. This was reasonable as we used less information. Generally, the results from the reduced models preserved all the observations we got from the full model, and only including the informatively collected labs did not yield significantly better results than only including regularly collected ones. However, the reduced models’ results gave some clearer interpretation when we only considered a subset of the variables. First, if we only incorporated the regularly collected measures, we saw that using missingness embedding and categorical encoding was more advantageous over other strategies in LSTM. Second, LOCF could greatly save us if we chose a poor strategy for a cross-sectional model like an MLP.

### Limitations

It is important to note that this study only investigated one prediction task using data from a single EHR system. The results may not fully generalizable. Performance outcomes may vary in different settings. However, it is valuable to repeat the study for various tasks using data from multiple EHR systems. This will allow us to further explore the external validity of our findings.

Model interpretability is a top priority in machine learning research. When the embedding technique is applied to numerous categories (e.g. word embeddings), the real-value embedding vectors can be visualized through their principal component projection in two dimensions on a scatter plot [34]. This visualization implies how various categories may relate to each other. However, in our scenario, the missingness embedding has two classes for each variable, making it difficult to envision their clinical significance using the above-mentioned method.

There is also room for improvement in obtaining embeddings. Our current approach generates constant embedding vectors for all missing values in each predictor or column. Namely, the vectors are only variable-specific. However, we can enhance this approach by incorporating time information in the embeddings and allowing them to be time-dependent. This will likely introduce more variability to the data but still adhere to the NMAR missingness mechanism.

Lastly, the metric used for performance evaluation needs adjustment. We used a weighted aggregated AUROC due to the longitudinal structure in the data, but for simplicity, we chose the proportions of the number of observations at each time block as the weights. This approach requires more justification. For future work, we need a metric that incorporates the repeated measure design.

## Conclusion

In this paper, we examined the different methods for handling informative missing data in EHR data. We found that our laboratory measurements follow an informative missingness pattern, where lab measurements were taken only when needed. To address it, we compared five missing data strategies combined with three downstream classifiers and found that the LSTM models generally outperformed other cross-sectional models. Our analysis revealed that LOCF combined with normal-value imputation or categorical encoding performed well under cross-sectional models, whereas missingness embedding and categorical encoding achieved similar performance and outperformed other approaches under LSTM models. One of the biggest advantages of missingness embedding was that it did not require prior knowledge of the data and can be used under any scenario to address missingness. In conclusion, we strongly recommend using an appropriate missing data method to avoid imposing incorrect assumptions. This is critical to prevent introducing bias and worsening the performance of predictive models.

## Data Availability

The datasets analyzed during the current study are not publicly available due to privacy but are available from the corresponding author on reasonable request.

## Abbreviations

EHR: Electronic health record
CPM: Clinical prediction model
MCAR: Missing completely at random
MAR: Missing at random
NMAR: Not missing at random
MI: Multiple imputation
EWS: Early warning score
ICU: Intensive care unit
LOCF: Last observation carried forward
LASSO: Least absolute shrinkage and selection operator
MLP: Multilayer perceptron
LSTM: Long short-term memory
AUROC: Area under receiver operating characteristics curve
RR: Relative risk
CI: Confidence interval
SMD: Standardized mean difference
